# Insight into the drug-resistant characteristics and genetic diversity of multidrug-resistant *Mycobacterium tuberculosis* in China

**DOI:** 10.1128/spectrum.01324-23

**Published:** 2023-09-21

**Authors:** Zexuan Song, Chunfa Liu, Wencong He, Shaojun Pei, Dongxin Liu, Xiaolong Cao, Yiting Wang, Ping He, Bing Zhao, Xichao Ou, Hui Xia, Shengfen Wang, Yanlin Zhao

**Affiliations:** 1 National Institute for Communicable Disease Control and Prevention, Chinese Center for Disease Control and Prevention, Beijing, China; 2 National Tuberculosis Reference Laboratory, Chinese Center for Disease Control and Prevention, Beijing, China; 3 School of Public Health, Peking University, Beijing, China; Johns Hopkins University School of Medicine, Baltimore, Maryland, USA

**Keywords:** *Mycobacterium tuberculosis*, multidrug resistance, whole genome sequence, compensatory mutations

## Abstract

Multidrug-resistant tuberculosis (MDR-TB) has a severe impact on public health. To investigate the drug-resistant profile, compensatory mutations and genetic variations among MDR-TB isolates, a total of 546 MDR-TB isolates from China underwent drug-susceptibility testing and whole genome sequencing for further analysis. The results showed that our isolates have a high rate of fluoroquinolone resistance (45.60%, 249/546) and a low proportion of conferring resistance to bedaquiline, clofazimine, linezolid, and delamanid. The majority of MDR-TB isolates (77.66%, 424/546) belong to Lineage 2.2.1, followed by Lineage 4.5 (6.41%, 35/546), and the Lineage 2 isolates have a strong association with pre-XDR/XDR-TB (*P* < 0.05) in our study. Epidemic success analysis using time-scaled haplotypic density (THD) showed that clustered isolates outperformed non-clustered isolates. Compensatory mutations happened in *rpoA*, *rpoC,* and non-RRDR of *rpoB* genes, which were found more frequently in clusters and were associated with the increase of THD index, suggesting that increased bacterial fitness was associated with MDR-TB transmission. In addition, the variants in resistance associated genes in MDR isolates are mainly focused on single nucleotide polymorphism mutations, and only a few genes have indel variants, such as *katG*, *ethA*. We also found some genes underwent indel variation correlated with the lineage and sub-lineage of isolates, suggesting the selective evolution of different lineage isolates. Thus, this analysis of the characterization and genetic diversity of MDR isolates would be helpful in developing effective strategies for treatment regimens and tailoring public interventions.

**IMPORTANCE:**

Multidrug-resistant tuberculosis (MDR-TB) is a serious obstacle to tuberculosis prevention and control in China. This study provides insight into the drug-resistant characteristics of MDR combined with phenotypic drug-susceptibility testing and whole genome sequencing. The compensatory mutations and epidemic success analysis were analyzed by time-scaled haplotypic density (THD) method, suggesting clustered isolates and compensatory mutations are associated with MDR-TB transmission. In addition, the insertion and deletion variants happened in some genes, which are associated with the lineage and sub-lineage of isolates, such as the *mpt64* gene. This study offered a valuable reference and increased understanding of MDR-TB in China, which could be crucial for achieving the objective of precision medicine in the prevention and treatment of MDR-TB.

## INTRODUCTION

Tuberculosis (TB), an infectious disease caused by *Mycobacterium tuberculosis* (*Mtb*), is a major health problem worldwide. Drug-resistant TB (DR-TB) remains a great challenge for TB control (1). WHO estimated almost half a million people have developed resistance to rifampicin (RR-TB), with three quarters of these cases being multidrug-resistant tuberculosis (MDR-TB, resistant to the first-line anti-tuberculosis drugs isoniazid and rifampicin), and underdiagnosis and treatment failures increase MDR-TB transmission and exacerbate the issue of drug resistant (2). Furthermore, people with MDR-TB require lengthier and more expensive treatment regimens, with lower cure rates and a higher risk of side effects from toxic drugs (3, 4).

Owing to the fact that phenotypic drug-susceptibility testing (pDST) is routinely performed by culture-based methods that require lengthy time and strict biosafety conditions, whole genome sequencing (WGS) has the potential to be a realistic way to diagnose the majority of drug-resistant cases by revealing genetic resistance (5). However, the efficacy of WGS as a diagnostic tool is entirely dependent on a complete and accurate catalog of drug-resistant mutations for each drug. To date, genotypic predictions of drug resistance correlate well with pDST results of *Mtb* against first-line drugs (6), but the mechanisms of the new and repurposed drugs are less well understood. As multidrug-resistant cases climb, the increased use of second-line and new drugs in the clinic highlights the need to investigate the genetic diversity of MDR isolates with diverse mutations in antibiotic resistance genes.

Genetic variation in *Mtb* isolates is dominated by single nucleotide polymorphisms (SNPs), followed by insertions and deletions (indels). Mutation in antibiotic resistance genes is generally characterized by single-nucleotide substitutions. Additionally, a previous study showed the role of indels in the evolution of antibiotic resistance (7), and a well-known example is the deletion of *katG* conferring isoniazid resistance. Furthermore, the long insertion sequence integrates the antibiotic resistance genes, such as IS6110 inserted into *Rv0678* (8). But the frequency and contribution of indel in the *Mtb* genome remain poorly understood. It is essential to focus on the role of indel in the genome, especially long sequences.

China is one of the high-burden countries worldwide for both TB and MDR-TB, and there are previous studies that reported the drug-resistant feature of MDR in local provinces of China (9 – 11), and the transmission of MDR isolates (10, 12). But few studies provide a comprehensive investigation of the drug-resistant genes and genetic diversity of MDR isolates at the population level. In this study, we could investigate the resistance characteristics and genetic diversity of MDR isolates of China in detail, promoting a thorough understanding of the processes underlying drug-resistant tuberculosis and generating efficient tuberculosis prevention and control strategies in China.

## MATERIALS AND METHODS

### The sample collection

**Fig 1:**
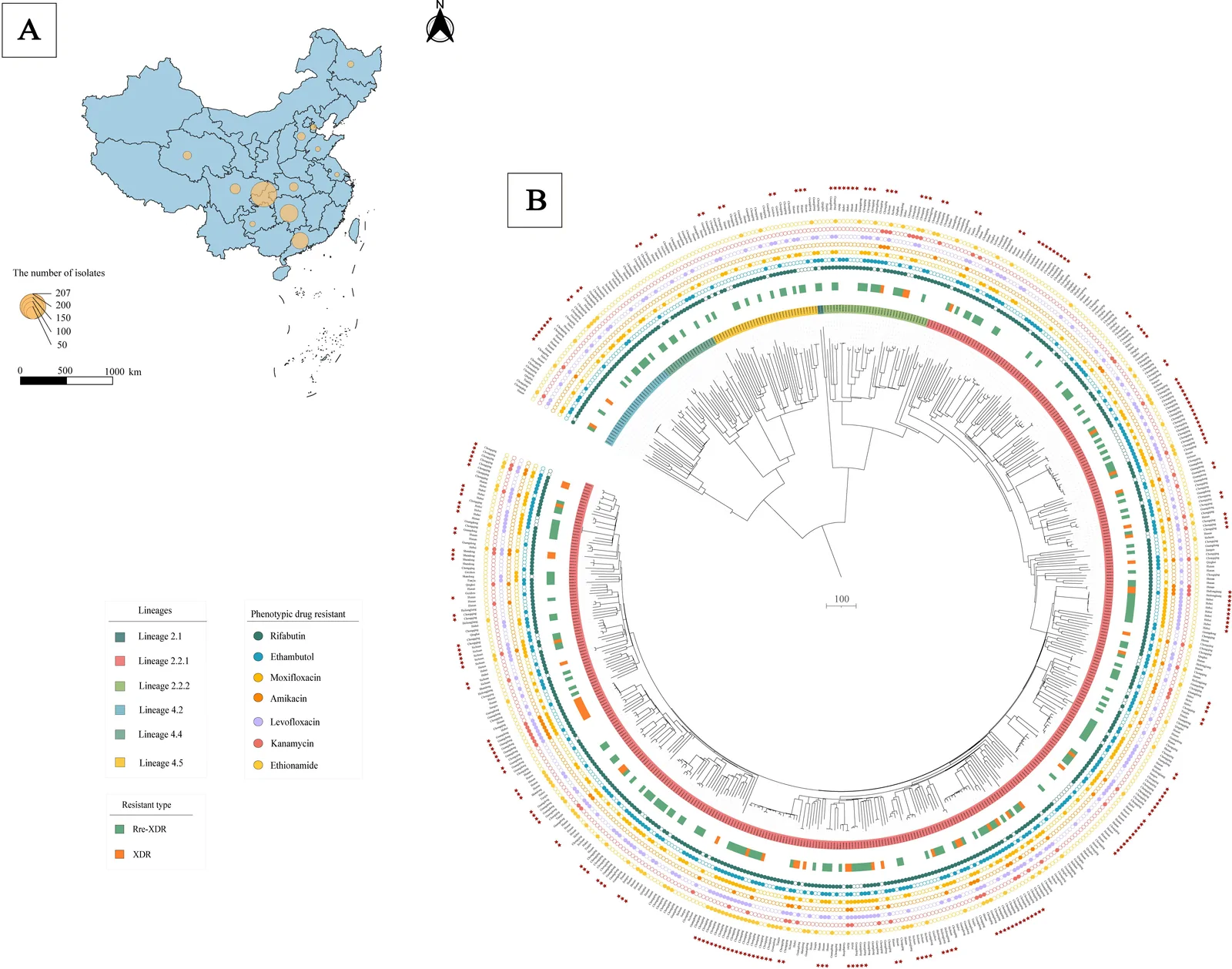
The phylogenetic structure of the MDR-TB isolates in this study. (**A**) The numbers of MTB isolates that were sampled from different geographic regions in China. (**B**) The maximum-likelihood phylogenetic tree of the 546 MDR isolates. The lineages, resistant type, phenotypic drug resistant and the geographic information of the isolates are shown (from inner to outer circles), according to the color legend shown on the left. The red stars indicating the clustered isolates are shown in the outermost ring.

In this study, 300 MDR-TB isolates (phenotypically resistant to rifampicin and isoniazid) were randomly selected from the National Tuberculosis Reference Laboratory (NTRL) in China, and 298 isolates were recovered successfully for follow-up studies. Subsequently, we included the 248 phenotypic MDR-TB isolates from the Hunan and Chongqing regions of our previous study to expand the sample size of the study (11, 13). The remaining 546 MDR-TB isolates were selected for in-depth analysis (Table S1), which collected from diverse geographic locations in China (Fig. 1A). The geolocation and the number of isolates were plotted on a map generated using QGIS (v3.30).

### Drug susceptibility testing

The drug-susceptibility testing (DST) of isolates was performed using UKMYC6 microdilution plate (Thermo Fisher, Scientific Inc., USA), which contain 5–10 doubling dilutions of 13 antibiotics (rifampicin, rifabutin, isoniazid, ethambutol, levofloxacin, moxifloxacin, amikacin, kanamycin, ethionamide, clofazimine, linezolid, delamanid, and bedaquiline). The DST was operated according to the standard operating protocol defined by CRyPTIC (14). Briefly, 0.5 McFarland suspensions of *Mtb* isolates prepared from fresh colonies (no longer than 14 d old) grown on Lowenstein-Jensen tubes were diluted 100-fold in 10 mL of 7H9 broth prior to plate inoculation. The semi-automated Sensititre Auto-inoculator was used to aliquot 100μL into each well of the UKMYC6 microdilution plate. Then all the plates were sealed and incubated for 14 d at 37°C. The DST results for each drug were separated by two trained laboratory operators using the Thermo Fisher Sensititre Vizion digital MIC viewing system. The minimum inhibitory concentration (MIC) is the lowest antibiotic concentration that inhibits observable microorganism growth. Quality control runs with reference *M. tuberculosis* H37Rv ATCC 27294 were performed on the plate on a regular basis. The concentration range and the breakpoint concentration of each drug included in this study have been shown in Table S2.

### Whole genome sequencing

All the MDR-TB isolates subculturing in Lowenstein-Jensen media were performed for DNA extraction using the cetyltrimethylammonium bromide (CTAB) method as previously described (15). The genomic DNA of every isolate was sequenced by Illumina Hiseq X Ten (Illumina, Inc.) with 2 × 150 bp pair-end reads. All the whole genome sequencing operations were completed by Annoroad Gene Technology company (Beijing, China).

### Bioinformatic analysis

The WGS analysis was carried out using a variant calling pipeline Clockwork developed by CRyPTIC (16). The quality control of the sequence reads was examined by FastQC (v0.11.9), then the reads were processed using the pipeline Clockwork with default parameters (v1.0). In the pipeline, the human, nasopharyngeal flora, and human immunodeficiency virus-related reads are removed and the remaining reads are trimmed (adapters and low-quality ends) using Trimmomatic software and mapped to the reference genome *M. tuberculosis* H37Rv (NC000962.3) with BWA-MEM. Genetic variants are called independently using Cortex and SAMtools. These two call sets are merged to produce a final call set by Minos. Then, the variants were annotated by snpEff (v5.0e). Structural variants relative to the reference H37Rv genome (NC_000962.3) were examined with Delly (v0.7.6) (17).

### Phylogenetic analysis

The SNPs located at known drug resistance-related genes, the mobile genetic elements, and PE or PPE regions were excluded from the phylogenetic analysis. Recombination core SNP alignment was constructed and a maximum likelihood phylogenetic tree was built by RAxML with 1,000 bootstrap replicates and a general time reversible (GTR + G) model of nucleotide substitution. The visualization and modification of the phylogenetic tree were performed by iTOL (v6.4.3). The isolates of lineage and sub-lineage were established by the fast-lineage-caller v1.0 (https://github.com/farhat-lab/fast-lineage-caller). And the python script snp-dists (v 0.8.2) was used to calculate the genomic pairwise distances (https://github.com/tseemann/snp-dists). The genomic cluster was defined as the isolates with genetic distance of 12 SNPs or less (18).

### Epidemic success analysis

The time-scaled haplotypic density (THD) success index was calculated using the R package THD as previously described (19, 20). The parameters employed were a mutation rate of 10^−7^ mutations per site per year, an effective genome size (number of positions retained for SNP calling) of 4.0 × 10^6^, and the time scale of 20 years.

### Statistical analysis

All statistical analysis was used with SPSS (v18.0). The χ^2^ test or Fisher’s exact test was performed for this study data. Differences in THD distribution across groups were tested using a two-sided Mann–Whitney U test. *P* value < 0.05 was considered statistically significant.

## RESULTS

### The population structure of MDR

To characterize the population structure and genetic diversity of MDR isolates, the phylogenetic tree was constructed based on core single nucleotide polymorphisms (SNPs) (Fig. 1B). Among the MDR isolates in our study, 84.25% (460/546) belonged to lineage 2 (L2) and 15.75% (86/546) were classified as lineage 4 (L4), suggesting L2 isolates are overrepresented in drug resistance isolates. Within the L2, the majority of isolates (92.17%, 424/460) belong to lineage 2.2.1, followed by lineage 2.2.2 (34/460) and lineage 2.1(2/460). And there were three sub-lineages of L4, which were lineage 4.2 (31/86), lineage 4.4 (19/86), and lineage 4.5(35/86). These findings are in line with the prior report that the lineage 2.2.1 isolates are more drugs resistant (21).

In the phylogenetic tree, we found a total of 231 isolates (42.31%, 231/546) were grouped into 70 genomic clusters, which ranged in size from 2 to 19 isolates. And the clustered rate was varied between the L2 and L4 (*x* ^2^ = 8.673, *P* = 0.003) (Table 1). To disentangle the respective influences of the cluster isolates and nonclusters on the transmission, we analyzed the THD success indices between the two groups. The results showed that isolates belonging to clusters displayed higher THD indices than noncluster isolates (Fig. 3A).

**TABLE 1:** Comparison of the clustering of isolates in lineages and CMs^*a*^

Classification	Clustered (%)	Non-clustered (%)	*χ* ^2^	*P*
Lineages				
Lineage 2	207 (45.0)	253 (55.0)	8.673	0.003
Lineage 4	24 (27.9)	62 (72.1)		
CMs				
With CMs	100 (52.0)	93 (49.0)	11.051	0.001
Without CMs	131 (36.9)	222 (63.1)		

^*a*^ CMs, the isolates with putative compensatory mutations.

### Drug-resistance characteristics of MDR

The drug-susceptibility testing for 13 anti-tuberculosis drugs was performed in MDR isolates, and resistance to any remaining 11 drugs was associated with resistance to both isoniazid and rifampicin (Table 2). The results show that MDR isolates were most commonly resistant to the first-line drug ethambutol (46.89%, 256/546), excluding rifabutin (84.07%, 459/546). Of the second-line drugs, levofloxacin and moxifloxacin more commonly have resistant phenotypes than the injectable drugs kanamycin and amikacin. For the fluoroquinolone resistance, 41.94% (229/546) and 40.48% (221/546) of the isolates are resistant to moxifloxacin and levofloxacin, respectively. And the percentage of MDR isolates with kanamycin resistant (12.45%, 68/546) is higher than amikacin resistant (10.99%, 60/546). However, the little proportion was resistant to bedaquiline (0.73%, 4/546), clofazimine (0.92%, 5/546), delamanid (0.92%, 5/546), and linezolid (1.09%, 6/546) in this study. The MICs distribution against bedaquiline, clofazimine, delamanid, and linezolid have been shown in Fig. S1. Of note, our results also showed that the isolates of lineage 2 were more likely to be resistant to ethambutol, levofloxacin, kanamycin, amikacin, and ethionamide than those of lineage 4 (*P* < 0.05) (Table 2).

**TABLE 2:** The drug resistance profiles of 546 MDR isolates in this study

Drugs	Total	Lineage 2	Lineage 4	*χ* ^2^	*P*
		No. (%)	No. (%)		
Ethambutol	256 (46.89%)	240 (52.17%)	16 (18.60%)	32.786	0.001
Moxifloxacin	229 (41.94%)	200 (43.48%)	29 (33.72%)	2.833	0.92
Levofloxacin	221 (40.48%)	196 (42.61%)	25 (29.07%)	5.512	0.019
Kanamycin	68 (12.45%)	66 (14.35%)	2 (2.33%)	9.605	0.002
Amikacin	60 (10.99%)	59 (12.83%)	1 (1.16%)	10.076	0.002
Ethionamide	116 (21.25%)	114 (24.78%)	2 (2.33%)	21.839	0.001
Rifabutin	459 (84.07%)	387 (84.13%)	72 (83.72%)	0.009	0.924
pre-XDR	249 (45.60%)	219 (47.61%)	30 (34.88%)	4.729	0.03
XDR	51 (9.34%)	49 (10.65%)	2 (2.33%)	5.932	0.015

According to previous definition of drug-resistant patterns, 45.60% (249/546) of MDR isolates were resistant to either any fluoroquinolone or any injectable drug belonging to pre-XDR-TB. Of the MDR isolates, 9.34% (51/546) had additional resistance to one fluoroquinolone (moxifloxacin or ofloxacin) and one injectable drug (kanamycin or amikacin), being referred to as XDR-TB (extensively drug resistant), with a higher percentage than reported globally (22). Indeed, the pre-XDR-TB and XDR isolates were significantly more likely to be present in lineage 2 in this study (Table 2).

### Genetic determinants of resistance

To understand the genetic mutations in MDR isolates, we proceeded to identify the mutation in Tier 1 candidate resistance genes in the recent WHO catalog (Table S3) (16). As expected, the common mutations linked to rifampicin resistance were *rpoB*_450, *rpoB*_445, and *rpoB*_435 codons. Of these mutations, S450L occurs most frequently, being present in 53.85% (294/546) of MDR isolates in this study. Among all mutant isolates, 33.33% (170/510) of them exhibited combined mutation in *rpoB*, and 46.47% (79/170) and 12.35% (21/170) of these combined with S450L and D435G, respectively. We also found that seven isolates exhibited D435F mutation, which means simultaneous mutations in two single nucleotide positions (1304_G > T and 1305_A > T). Of note, there are 29 isolates which exhibit combined mutation in Rifampicin Resistance Determining Region (RRDR). We also found five isolates exhibited an indel variant, which is rare in the reports (Table S4).

In addition, 71.25% (389/546) of MDR isolates had *katG*_S315T mutation, which is the primary mutation for isoniazid resistance. 83.34% (455/546) of the isolates exhibited *katG* R463L mutation. Except for one isolate is lineage 4, all isolates belong to lineage 2. We also found that in 1.74% (9/517) isolates,a premature stop codon mutation occurred, and in 5.42% (28/517) isolates, an indel variant in *katG* gene occurred, which could affect the ability of KatG enzyme and cause isoniazid resistance. Additionally, 12.96% (67/517) of mutation in *katG* combined with *ahpC* promoter mutations in our study (Table S5). But it is not yet clear whether *ahpC* promoter mutations have a compensatory effect on KatG activity (23).

Among 256 MDR isolates with phenotypically ethambutol resistance, the main mutation was *embB*_M306V (38.67%, 99/256), followed by M306I (19.14%,49/256). We found that mutations *embB* _G406V, *embB* _Q853E, and *embB* _ Y319D occurred in 2, 2 and 1 isolates, respectively, which are not included in the tb-profiler database and the function needs to be further investigated. Conversely, the *embB* D1024N variant contained in the tb-profiler database, while 10 isolates in this study containing this single mutation were not resistant to ethambutol. We also found that there was no indel mutation in *embB* gene (Table S6). The fluoroquinolone-resistant mutations are predominant in *gyrA*, which encodes the gyrase A subunit. gyrA_D94G and gyrA_A90V were the two often occurring mutations for moxifloxacin and levofloxacin (Tables S7 and S8), and even two isolates had this double mutation in this study. Additionally, 70.59% (48/68) of kanamycin-resistant isolates and 76.67% (38/60) of amikacin-resistant isolates carried a-1401 g mutation in *rrs* gene (Tables S9 and S10). For ethionamide resistance isolates, the top mutation was c-15t in *fabG1* (41.38%, 48/116), and 26.7% (31/116) isolates happened indel mutation in *ethA* (Table S11).

We also determined the mutation associated with resistant to new and repurposed drugs (bedaquiline, delamanid, linezolid and clofazimine), but with low phenotypic resistance rates to these drugs. Mutations *Rv0678*_L117R and *Rv0678_*R134* were both detected in one bedaquiline-resistant isolate. Mutation *rplC* _C154R was found in four linezolid-resistant isolates. However, no know mutations associated with clofazimine and delamanid were detected in resistant isolates.

### Compensatory mutations analysis

**Fig 2:**
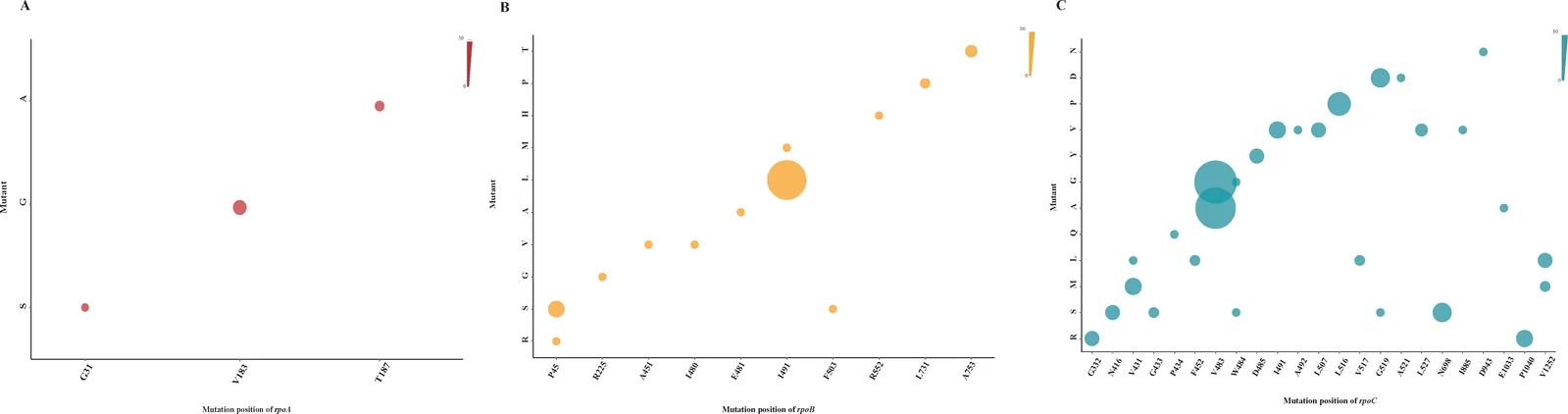
Putative compensatory mutations in the *rpoA*, *rpoB*, and *rpoC* genes were identified in this study. Each putative compensatory mutation was supported by at least two independent evolution events on the phylogenetic tree.

To determine the putative compensatory mutations (CM) in MDR isolates, the variants in three compensatory target genes (*rpoA*, *rpoC,* and *rpoB* compensate fitness effects of *rpoB* mutations in rifampicin resistant) were investigated. By plotting the mutations onto the ML phylogeny tree, we identified 36 codons with putative CMs (Fig. 2), which occurred independently at least twice in our study. Of these codons, only about 8.33% (3/36) occured in *rpoA*, but the majority occured in *rpoC* (63.89%, 23/36), which is consistent with the previous study (23). In total, 35.35% (193/546) of the isolates in this study carried putative CMs; the most commonly putative CM is *rpoC*_V483G (9.33%, 18/193), followed by *rpoC*_V483A (8.81%, 17/193) and *rpoB_*I491L (8.81%, 17/193). Unlike the previously reported compensatory mutation *rpoB*_I491V, this study showed two mutations (I491L and I491M) with putative compensatory function at the same codon 491 of *rpoB*. In addition, some isolates also exhibited CM mutation outside of the RRDR in this study, such as *rpoB*_P45S, *rpoB*_P45L, and *rpoB*_I480V, which could compensate for the growth deficits caused by mutation *rpoB*_S450L to varying degrees (24).

**Fig 3:**
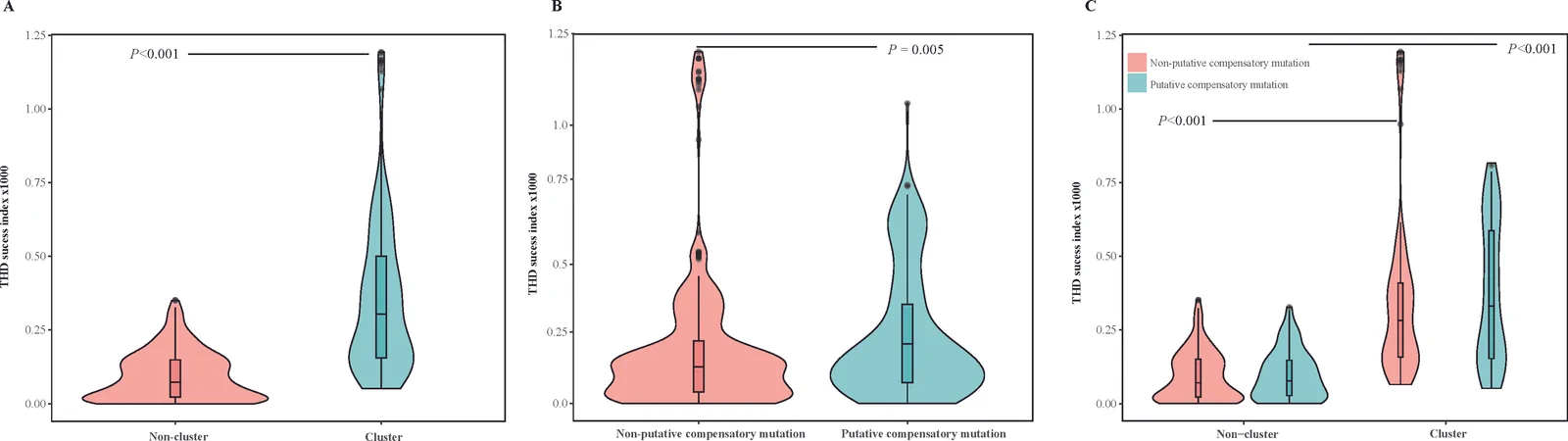
The influences of cluster and putative compensatory mutations on THD success indices of the MDR isolates in this study. *P*-values obtained from 2-sided Mann–Whitney U test.

To date, there have been inconsistent findings on the effect of CM on the transmission of MDR isolates (25, 26). In this study, we found there are statistically significant differences in the ratios of putative compensatory mutations between clustered and non-clustered MDR-TB isolates (*x* ^2^ = 11.051, *P* = 0.001) (Table 1). In addition, we compared THD success index across the CM and non-CM isolates in this study (Fig. 3B), which could reflect the influence of the putative compensatory mutations on the transmission success of MDR isolates. The results showed that isolates with putative compensatory mutations outperformed the non-compensatory mutation isolates (*P* = 0.005). Nevertheless, the presence of putative compensatory mutations was not associated with increased THD indices in cluster or non-cluster isolates (Fig. 3C).

### Insertions and deletions in genes of MDR isolates

The previous study showed that insertion and deletions (indels) are important contributors to *M. tuberculosis* evolution (7). Indels were common in PE-PPE genes; however, because of the low sequence complexity of these genes, indel-calling mistakes can increase, lowering the precision of this estimate (27). Thus, we investigated the intra-genic insertion and deletion mutations excluding PE-PPE genes in this study. There are 21 genes that exhibited indel mutation in more than 10 isolates (Table 3). Interestingly, we found all isolates of lineage 4.2.2 harbored 63 bp deletion in *mpt64* genes, which encodes the secreted protein MPT64 and it was employed as a specific antigen for MTBC detection (28). There were 425 isolates happened 35-bp insertion in *espK* gene, which encodes EspK protein with an active role in ESX-1-mediated secretion (29). These isolates were widely distributed in lineage 2.1, lineage 2.2.1, lineage 2.2.2, and lineage 4.2. We also noted that 27 isolates have 55 bp deletion in the *eccC2* genes, which could influence the activity of the ESX-1 system. These isolates all belong to a clade of the lineage L2.2.1. In addition, the genes *fhaA*, *fadD34*, *Rv0063*, *Rv0140*, *Rv0538*, *Rv0559c*, *fbiC*, *Rv1435c*, *Rv1667c*, *Rv1730*, *Rv1883c*, *Rv2006*, *qcrB*, *Rv2407*, *Rv2434c*, *mmuM*, *Rv3080c*, *accE5,* and *Rv3785* have varying degree of indels in this study, but their functions need to be further investigated.

**TABLE 3:** The insertion and deletion variance in genes (excluding PE and PPE) of MDR isolates in this study

Gene name	Genetic changes position			Gene function	The number of the isolates	The lineages of isolates
	Position	Type	Length			
*fhaA*(Rv0020c)	24698	DEL	18 bp	Signal transduction	22	L2.2.1(20), L2.2.2(2)
*fadD34*(Rv0035)	37553	INS	21 bp	Function unknown, but involved in lipid degradation	19	L4.4(19)
Rv0063	67070	INS	29 bp	probably involved in cellular metabolism	70	L2.2.1(70)
Rv0140	167144	DEL	369 bp	Function unknown	12	L2.2.1(12)
Rv0538	631326	DEL	72 bp, 36 bp	Unknown	31	L4.2(30), L4.5(1)
Rv0559c	650639	DEL	29 bp	Unknown	14	L2.2.1(14)
*fbiC*(Rv1173)	1305494	DEL	124 bp, 62 bp	Essential for coenzyme F420 production	52	L2.2.1(13), L2.2.2(1), L4.2(2), L4.2(3), L4.5(33)
Rv1435c	1612624	INS	21 bp	Function unknown	52	L2.2.1(39), L2.2.2(1), L4.2(7), L4.4(2), L4.5(3)
Rv1730	1955913	DEL	18 bp	Thought to be involved in cell wall biosynthesis and may also act as a sensor of external penicillins	305	L2.2.1(305)
Rv1883c	2133468	INS	15 bp, 17 bp, 46 bp	Function unknown	478	L2.1(2), L2.2.1(374), L2.2.2(28), L4.2(30), L4.4(19), L4.5(25)
*mpt64*(Rv1980c)	2223770	DEL	63 bp	Immunogenic protein Mpt64 (antigen Mpt64/MPB64)	27	L4.2(27)
Rv2006	2255904	INS	20 bp	Probable trehalose-6-phosphate phosphatase OtsB1	32	L4.5(32)
*qcrB*(Rv2196)	2461325	DEL	114 bp, 57 bp	ubiquinol-cytochrome C reductase cytochrome subunit B	39	L2.2.1(3), L4.2(2), L4.5(34)
Rv2407	2704884	INS	21 bp	Function unknown	514	L2.1(2), L2.2.1(401), L2.2.2(30), L4.2(31), L4.4(16), L4.5(34)
Rv2434c	2729618	DEL	214 bp	Unknown	273	L2.2.1(273)
*mmuM*(Rv2458)	2760249	DEL	141 bp	Catalyzes methyl transfer from S-methylmethionine or S-adenosylmethionine (less efficient) to homocysteine, selenohomocysteine, and less efficiently selenocysteine	12	L2.2.1(12)
*pknK*(Rv3080c）	3443716	DEL	26 bp	Involved in signal transduction	14	L2.2.1(14)
*accE5*(Rv3281)	3663727	DEL	198 bp, 135 bp	Involved in long-chain fatty acid synthesis	355	L2.2.1(334), L2.2.2(21)
Rv3785	4231859	DEL	89 bp	Unknown	456	L2.1(2), L2.2.1(422), L2.2.2(32)
*espK*(Rv3879c)	4359163	INS	35 bp	ESX-1 secretion-associated protein EspK	425	L2.1(1), L2.2.1(373), L2.2.2(29), L4.2(22)
*eccC2*(Rv3894c)	4377269	DEL	55 bp	ESX-2 type VII secretion system protein	27	L2.2.1(27)

## DISCUSSION

This study further reveals the genetic diversity and drug-resistance profile of MDR isolates in China. As expected, Lineage 2, especially the sub-lineage L2.2.1, was the dominant genotype of the MDR isolates. Previous studies showed that Beijing genotype (L2) is strongly associated with drug resistance (11, 30), and our study provides evidence that Beijing genotype isolates are more resistant to ethambutol, levofloxacin, kanamycin, amikacin, and ethionamide. This result may be related to the various mutation rates of drug resistance in different lineages. Lineage 2 isolates, in particulat, have been proposed to acquire drug resistance more rapidly *in vitro* (31). However, Zhao et al. previously reported that the prevalence of drug resistance was higher in Beijing genotype than in non-Beijing genotype isolates, but there was no significant difference between these two genotypes in multivariate analysis (32). The conflicting result might be due to the proportion of the Beijing genotype and its sub-lineages (lineage 2.2.1 and lineage 2.2.2), as this can impact the pDST results and subsequent statistical analysis. In addition, differences in the geographic settings of isolates, treatment regimens, and patient compliance may also influence the results of drug resistance analysis.

Our data show the high percentage of pre-XDR and XDR isolates among the MDR isolates population in this study. According to the old definition, 45.60% and 9.34% of the MDR isolates in our study were pre-XDR and XDR, respectively. The strong correlation was shown between L2 isolates and pre-XDR/XDR-TB. The striking shift toward pre-XDR was mediated by a substantial rate of fluoroquinolone resistance. The rate of MDR-TB resistance to fluoroquinolones in our study was significantly higher than that of the national survey conducted in 2008 (33), which was supported by the recent report (34). The rate of XDR in our study is higher than the previous global report, which estimates that about 6% of MDR-TB patients have XDR-TB infection (35). To date, fluoroquinolones will remain the mainstay of MDR-TB treatment in China until new medications and regimens become widely accessible, which emphasizes the urgent necessity for fluoroquinolone resistance diagnosis before developing a treatment regimen. We noted the low proportions of our isolates conferring resistance to bedaquiline, clofazimine, linezolid, and delamanid, suggesting that these drugs are effective in the treatment of MDR-TB.

Gene variants associated with drug resistance were further investigated. In keeping with earlier studies, the rifampicin resistance-determining region (RRDR) and *katG* 315 codon mutations were clearly predominate in the examination of rifampicin and isoniazid-resistant mutations. Numerous publications have noted that isoniazid-resistant isolates carry mutation R463L, although this mutation is not sufficient to indicate isoniazid resistance (36, 37). The mutation was shown to be strongly associated with lineage 2 in this study, suggesting that the variant may have arisen as a result of selection pressure on various lineage strains. The idea that this mutation is linked to drug resistance may be due to the high percentage of lineage 2 strains in most studies investigating drug resistance. Of note, there were some isolates with phenotypic resistance against anti-TB drugs for the mutations in these genes could not be detected, suggesting alternative mechanisms, such as drug efflux pump.

Previous studies showed that CM could partially or completely restore the fitness cost of the drug resistance conferring mutations (23, 24). Whereas numerous research studies have attempted to determine how CMs affected the spread of MDR-TB, the findings were inconsistent (26, 38, 39). In our study, MDR isolates with putative CMs are more frequently found in clusters, suggesting increased bacterial fitness is associated with the transmission of MDR-TB. This result is supported by a recent report by Gygli et al., which revealed that CMs in the RNA polymerase of *M. tuberculosis* contribute to the transmission fitness of MDR-TB (39). However, Liu et al. found that MDR isolates with CMs in the RNA polymerase genes were not more frequently clustered than those without CMs (26). CMs in the non-RRDR region of *rpoB*, as well as those in *rpoA* and *rpoC*, were found in our study, which show that the *rpoB* non-RRDR mutations may have compensatory effects (24). Notably, previous studies have shown the mutation with compensatory (I491V) and resistance function (I491F) at codon 491 of *rpoB* (24, 40), while some isolates had mutations in *rpoB*_I491L and *rpoB*_I491M distributed on different evolutionary branches in this study, so they were tentatively considered as putative compensatory mutations, and the specific functions need to be confirmed by further studies. We also found that several mutations were shared by numerous isolates and evolved independently multiple times, suggesting a significant selection advantage for MDR isolates harboring these mutations. We investigated the putative CMs, predominate in *rpoC*, which may provide molecular indicators to predict highly adapted drug-resistant strains.

To date, the insertions and deletions to *M. tuberculosis* genome remain poorly understood, especially within genes. We all know that MPT64 protein is used to identify MTBC with high sensitivity and specificity (41), but the 63 bp deletion of *mpt64* could have a significant impact on the diagnosis of MTBC, resulting in false-negative results (42). To date, the 63bp deletion in *mpt64* had previously been reported in some clinical strains (43), but this variant is not common and no correlation with the lineage of isolates has been found. Our study showed 4.9% (27/546) of the isolates exhibited 63 bp deletion in *mpt64* genes and all isolates belong to lineage 4.2.2, suggesting this variation may have a strong correlation with lineage 4.2.2, and some L4.2.2 isolates may not be accurately diagnosed based on MPT64 assay in clinical. In addition, there are 425 isolates that exhibited 35 bp insertion mutation in *espK*, and this mutation occurred in mostly isolates of lineage 2 and lineage 4.2 (Table 3). Lim et al. demonstrated that EspK is needed for EsxA and EspB secretion and is an active component of the ESX-1 secretion machinery for *M. tuberculosis* (29). However, this mutation is the result of natural evolution, and further knockout and complementation research are required to determine whether it has an effect on the Espk function.

There are several limitations in our study. First, although our isolates covered 12 provinces, the isolates were highly skewed toward Chongqing, Hunan, and Guangdong regions, with fewer samples from other regions. Therefore, there may be some limitations in the representativeness of the samples for the entirety of China. Second, this study mainly focused on the drug resistance and genetic variation of MDR isolates, lacking epidemiological information on the isolates; thus, the transmission of the MDR isolates has not been thoroughly and comprehensively investigated. And the insertion and deletion variants only in MDR strains were studied, which are not sufficiently representative of genomic variation in the genome of *M. tuberculosis*.

In conclusion, the high-rate fluoroquinolones resistance of MDR isolates demand serious attention, underscoring the urgent need for identifying fluoroquinolones resistance prior to establishing a treatment regimen. Our study showed that the MDR isolates that acquitted putative compensatory mutations were accompanied by an increase in their THD success index. Our data also indicated that the variants in resistance-associated genes in MDR isolates are mainly focused on SNP mutations, with indel variants in only a few genes, such as *katG*, *ethA*. Furthermore, we found that some genes underwent indel variations. While there seems to be some correlation with the lineage and sub-lineage, this suggests a correlation with the evolution of the isolates, a function that requires further research.

## Data Availability

Sequencing reads have been submitted to the Sequence Read Archive (SRA) under the accession number PRJNA987438.

## SUPPLEMENTAL MATERIAL

Tables S2 to S11 spectrum.01324-23-s0001.docx Table S2: The concentration range and critical concentration of 13 anti-tuberculosis drugs. Table S3: Candidate genes determined to be associated with phenotypic resistance to the drugs. Table S4: The distribution of gene mutations associated with rifampicin resistance. Table S5: The distribution of gene mutations associated with isoniazid resistance. Table S6: The distribution of gene mutations associated with ethambutol resistance. Table S7: The distribution of gene mutations associated with moxifloxacin resistance. Table S8: The distribution of gene mutations associated with levofloxacin resistance. Table S9: The distribution of gene mutations associated with amikacin resistance. Table S10: The distribution of gene mutations associated with kanamycin resistance. Table S11: The distribution of gene mutations associated with ethionamide resistance.

Figure S1 spectrum.01324-23-s0002.pdf The MIC distribution of the 546 MDR isolates against bedaquiline, clofazimine, delamanid, and linezolid. Purple indicates drug-resistant strains with MIC greater than the breakpoint.

Table S1 spectrum.01324-23-s0003.xlsx The detailed information on 546 MDR isolates used in this study.

ASM does not own the copyrights to Supplemental Material that may be linked to, or accessed through, an article. The authors have granted ASM a non-exclusive, world-wide license to publish the Supplemental Material files. Please contact the corresponding author directly for reuse.
