# Supplementary material for: Insight into the drug-resistant characteristics and genetic diversity of multidrug-resistant Mycobacterium tuberculosis in China
Source: Microbiol Spectr. 2023 Sep 21;11(5):e01324-23. doi: 10.1128/spectrum.01324-23 (PMC10581218; doi:10.1128/spectrum.01324-23)
Supplement: Tables S2 to S11 — Table S2: The concentration range and critical concentration of 13 anti-tuberculosis drugs. Table S3: Candidate genes determined to be associated with phenotypic resistance to the drugs. Table S4: The distribution of gene mutations associated with rifampicin resistance. Table S5: The distribution of gene mutations associated with isoniazid resistance. Table S6: The distribution of gene mutations associated with ethambutol resistance. Table S7: The distribution of gene mutations associated with moxifloxacin resistance. Table S8: The distribution of gene mutations associated with levofloxacin resistance. Table S9: The distribution of gene mutations associated with amikacin resistance. Table S10: The distribution of gene mutations associated with kanamycin resistance. Table S11: The distribution of gene mutations associated with ethionamide resistance. [file spectrum.01324-23-s0001.docx]

**Table S2. The concentration range and critical concentration of 13 anti-tuberculosis drugs.**

| Drug | Concentration range(μg/mL) | Critical concentration (μg/mL) |
| --- | --- | --- |
| Isoniazid | 0.025-12.8 | 0.2 |
| Rifampicin | 0.03-8 | 1 |
| Rifabutin | 0.06-2 | 0.5 |
| Ethambutol | 0.25-32 | 5 |
| Ethionamide | 0.25-8 | 5 |
| Levofloxacin | 0.12-8 | 1 |
| Moxifloxacin | 0.06-4 | 0.5 |
| Amikacin | 0.25-16 | 4 |
| Kanamycin | 1-16 | 5 |
| Bedaquiline | 0.008-1 | 0.12 |
| Clofazimine | 0.03-2 | 1 |
| Delamanid | 0.008-0.5 | 0.12 |
| Linezolid | 0.06-4 | 1 |

**Table S3. Candidate genes determined to be associated with phenotypic resistance to the drugs.**

| Genes | Rv number | Star | Stop | Length | Associated antibiotic resistance |
| --- | --- | --- | --- | --- | --- |
| *ahpC* | Rv2428 | 2726193 | 2726780 | 588 | isoniazid |
| *inhA* | Rv1484 | 1674202 | 1675011 | 810 | isoniazid, ethionamide |
| *katG* | Rv1908c | 2153889 | 2156111 | 2223 | isoniazid |
| *rpoB* | Rv0667 | 759807 | 763325 | 3519 | rifampicin, rifabutin |
| *rpoA* | Rv3457c | 3877464 | 3878507 | 1044 | rifampicin, rifabutin |
| rpoC | Rv0668 | 763370 | 767320 | 3951 | rifampicin, rifabutin |
| embA | Rv3794 | 4243233 | 4246517 | 3285 | ethambutol |
| embB | Rv3795 | 4246514 | 4249810 | 3297 | ethambutol |
| *embC* | Rv3793 | 4239863 | 4243147 | 3285 | ethambutol |
| *gyrB* | Rv0005 | 5240 | 7267 | 2028 | moxifloxacin, ofloxacin, levofloxacin |
| *gyrA* | Rv0006 | 7302 | 9818 | 2517 | moxifloxacin, ofloxacin, levofloxacin |
| *pepQ* | Rv2535c | 2859300 | 2860418 | 1119 | bedaquiline, clofazimine |
| *Rv0678* | Rv0678 | 778990 | 779487 | 498 | bedaquiline, clofazimine |
| *mmpL5* | Rv0676c | 775586 | 778480 | 2895 | bedaquiline, clofazimine |
| *mmpS5* | Rv0677c | 778477 | 778905 | 429 | bedaquiline, clofazimine |
| *atpE* | Rv1305 | 1461045 | 1461290 | 246 | bedaquiline |
| *rplC* | Rv0701 | 800809 | 801462 | 654 | linezolid |
| *rrl* | Rvnr02 | 1473658 | 1476795 | 3138 | linezolid |
| *fgd1* | Rv0407 | 490783 | 491793 | 1011 | delamanid, PA-824 |
| *ddn* | Rv3547 | 3986844 | 3987299 | 456 | delamanid, PA-824 |
| *fbiA* | Rv3261 | 3640543 | 3641538 | 996 | delamanid, PA-824 |
| *fbiB* | Rv3262 | 3641535 | 3642881 | 1347 | delamanid, PA-824 |
| *fbiC* | Rv1173 | 1302931 | 1305501 | 2571 | delamanid, PA-824 |
| *Rv2983* | Rv2983 | 3339118 | 3339762 | 645 | delamanid, PA-824 |
| *rrs* | Rvnr01 | 1471846 | 1473382 | 1537 | amikacin, kanamycin |
| *eis* | Rv2416c | 2714124 | 2715332 | 1209 | amikacin, kanamycin |
| *whiB7* | Rv3197A | 3568401 | 3568679 | 279 | amikacin, kanamycin |
| *ethA* | Rv3854c | 4326004 | 4327473 | 1470 | ethionamide |

| **Table S4 The distribution of gene mutations associated with rifampicin resistance.** | |
| --- | --- |
| *rpoB* mutation  (codon position) | No. of isolates with  resistant phenotype |
| S450L; | 215 |
| H445T; | 34 |
| S450L;A286V; | 19 |
| H445D; | 18 |
| D435V; | 17 |
| D435G;I491L; | 14 |
| L452P; | 9 |
| H445L; | 7 |
| D435F; | 7 |
| S450L;P45S; | 6 |
| S450F; | 6 |
| D435G;L430P; | 5 |
| S450L;V183G; | 5 |
| H445L;P969S; | 4 |
| S441Q; | 4 |
| D435V;T676A; | 3 |
| H445R; | 3 |
| L43P;H445Q; | 3 |
| LS430PG; | 3 |
| S450L;L731P; | 3 |
| S450W; | 3 |
| D435A;R448Q; | 2 |
| D435G | 2 |
| D435T; | 2 |
| D435T;N437D;A451V; | 2 |
| Q172R;F424V;S672F; | 2 |
| Q172R;V170A;P45R; | 2 |
| E481A;P483L; | 2 |
| E81G;S450L; | 2 |
| H445N;L430P; | 2 |
| H445N;L452P; | 2 |
| H445N;V168M; | 2 |
| I491F; | 2 |
| L430P;N437D; | 2 |
| L430P;I491L; | 2 |
| L452P;M434I; | 2 |
| S441L; | 2 |
| S450L;A753T; | 2 |
| S450L;R225G; | 2 |
| S450L;R552H; | 2 |
| S450L;D631G; | 2 |
| S450L;G915S; | 2 |
| S450L;I48V; | 2 |
| S450L;F53S; | 2 |
| S450L;P45R; | 2 |
| S450L;T58P; | 2 |
| S450L;V523L; | 2 |
| D435E;S441L; | 1 |
| D435G;R448Q; | 1 |
| D435G;L430R; | 1 |
| D435T;I491L; | 1 |
| D435V;F433S; | 1 |
| D435V;P454S; | 1 |
| D435V;T427A; | 1 |
| c.1297_1299dupTTC | 1 |
| c.1297_1302dupTTCATG | 1 |
| c.1301_1303delTGG | 1 |
| c.1328_1336delTGACCCACAinsGGCCCC | 1 |
| Q172R;D435T; | 1 |
| Q382R;P28S;S441L; | 1 |
| Q432R;P321T;S450A; | 1 |
| Q432K; | 1 |
| Q432P; | 1 |
| H445R;R67S; | 1 |
| H445R;D92G;G633D; | 1 |
| H445R;I135T; | 1 |
| H445N; | 1 |
| H445N;Q172H; | 1 |
| H445N;G442R; | 1 |
| H445N;V168A; | 1 |
| H445C; | 1 |
| H445L;D435E; | 1 |
| H445T;L378R; | 1 |
| H445T;V170F; | 1 |
| L430P; | 1 |
| L430P;A584G;F424V; | 1 |
| L430P;D435A; | 1 |
| L430P;D435A;N437H; | 1 |
| L430P;H445R; | 1 |
| L430P;F424V; | 1 |
| L430P;S493T; | 1 |
| L430P;V170A; | 1 |
| L430P;V170A;R827C; | 1 |
| L452P;A753T; | 1 |
| L452P;H445Q; | 1 |
| L452P;H674R; | 1 |
| L452P;P7L; | 1 |
| L452P;V170A; | 1 |
| K83E;V170F; | 1 |
| K891T;S45M; | 1 |
| MD434IG; | 1 |
| P439A;c.1296_1307delATTCATGGACCA | 1 |
| S441L;K446R; | 1 |
| S450L;A286V;D253G; | 1 |
| S450L;A753V;E24K; | 1 |
| S450L;R18C; | 1 |
| S450L;R552C; | 1 |
| S450L;R827C; | 1 |
| S450L;N769T; | 1 |
| S450L;D545E; | 1 |
| S450L;Q41R; | 1 |
| S450L;E563G; | 1 |
| S450L;G376C; | 1 |
| S450L;G633S; | 1 |
| S450L;G798S; | 1 |
| S450L;H533N; | 1 |
| S450L;I491V; | 1 |
| S450L;I588V; | 1 |
| S450L;K891E; | 1 |
| S450L;P45L; | 1 |
| S450L;S567P; | 1 |
| S450L;T159P; | 1 |
| S450L;T400A; | 1 |
| S450L;T444A; | 1 |
| S450L;T756A; | 1 |
| S450L;V534M; | 1 |
| S450L;V562G; | 1 |
| S45W;G1000S; | 1 |
| TH444ST; | 1 |
| V170F; | 1 |
| V170F;H835R; | 1 |
| V170F;I488V; | 1 |
| WT | 31 |
| total | 546 |

| **Table S5 The distribution of gene mutations associated with isoniazid resistance.** | | | |
| --- | --- | --- | --- |
| *katG* mutation  (codon position) | *ahpC* mutation  (codon position) | *inhA* mutation  (codon position) | No. of isolates with resistant phenotype |
| S315T; |  |  | 334 |
| S315T; |  | c.-15C>T | 11 |
| S315N; |  |  | 10 |
| S315T; |  | c.-8T>C, | 8 |
| S315T; |  | c.-8T>C | 7 |
| S315N; |  | A81E; | 5 |
|  |  | c.-15C>T | 4 |
| c.974_975insCC(T326fs;) | c.-74G>A |  | 3 |
| R249G; |  | L203L | 3 |
| c.446G>A(W149*;) | c.-48G>A |  | 2 |
| Chromosome:g.2153889_2156147del | c.-54C>T |  | 2 |
| R146G; |  |  | 2 |
| D94G; |  |  | 2 |
|  |  | c.-15C>T;I21T | 2 |
| I228L; | c.-15C>T |  | 2 |
| S315T; | G-158C; |  | 2 |
| S315T; | G-308A; | c.-8T>C, | 2 |
| S315T; |  | L203L | 2 |
| S315T;T625A; |  |  | 2 |
| S315T;V697L; |  |  | 2 |
| Y337C;R425G; | c.G-51A; |  | 2 |
| V1A; |  |  | 2 |
| Chromosome:g.2155413_2155713del | c.-48G>A |  | 1 |
| c.1063delG(A355fs;) | c.-48G>A |  | 1 |
| c.1033A>T(K345*) | c.-52C>T |  | 1 |
| c.272G>A(W91*) | c.-52C>T |  | 1 |
| c.1144_1145insC(L382fs;) | c.-54C>T |  | 1 |
| c.1967_1976del, (D656fs;) | c.-54C>T |  | 1 |
| c.446G>A(W149*) | c.-54C>T |  | 1 |
| Chromosome:g.2154631_2154973del | c.-72C>T |  | 1 |
| c.1236G>A(W412*) | c.-74G>A |  | 1 |
| c.1432_1432del(A478fs;) | c.-81C>T |  | 1 |
| A109T; |  | c.-15C>T;I194T | 1 |
| A379T;T344P; |  | c.-15C>T | 1 |
| A411D; | c.-48G>A |  | 1 |
| A492D; | c.-57C>T |  | 1 |
| R249S;D656G; |  |  | 1 |
| R498S; |  |  | 1 |
| N138D; |  |  | 1 |
| N508D; |  | c.-15C>T | 1 |
| D329N;T112I; |  |  | 1 |
| D419A; |  | c.-15C>T | 1 |
| D735A; |  | c.-52C>T | 1 |
| c.2221T>C(Ter741Rext*) |  |  | 1 |
| c.359_360insCGGCGCCGGGGGCGG(G120_G121insGAGGG;) |  |  | 1 |
| katG_p.M257T |  | c.-15C>T | 1 |
| I95L; |  | c.-15C>T | 1 |
| Q127P; |  | c.-15C>T | 1 |
| E294G; |  |  | 1 |
| G125C;, |  | c.-15C>T;V92M; | 1 |
| G169S; |  | c.-15C>T | 1 |
| G268S; |  |  | 1 |
| G299V;, | c.-81C>T;c.-74G>A |  | 1 |
| G629D; | c.G-51A; |  | 1 |
| H270P; | c.-57C>T |  | 1 |
| I389L;Chromosome:g.2155678_2156147del, | c.-52C>T |  | 1 |
|  |  | S94A | 1 |
| c.1079_1079del(G360fs;) | 47insT |  | 1 |
| c.1245_1246insG, (D695fs;) | c.-81C>T |  | 1 |
| c.146_147insTGCA, (Q50fs;) | c.-57C>T |  | 1 |
| c.1574_1575insT, (Q525fs;) | c.-57C>T |  | 1 |
| c.176_176del(G59fs;) | c.-52C>T |  | 1 |
| c.1856_1857insT, (S620fs;) | c.-81C>T |  | 1 |
| c.2083_2084insCGTGG,;(D695fs;) | G-158C |  | 1 |
| c.2086_2086del, (L696fs;) | c.-48G>A |  | 1 |
| c.285_292del(Y95fs;) | c.-52C>T |  | 1 |
| c.371_371del(G124fs;) |  |  | 1 |
| Chromosome:g.2153889_2156147del |  |  | 1 |
| Chromosome:g.2153889_2156147del, | c.-52C>T |  | 1 |
| Chromosome:g.2153889_2156147del, | c.-57C>T |  | 1 |
| Chromosome:g.2154631_2154973del, | c.-72C>T |  | 1 |
| Chromosome:g.2155811_2156147del, | c.-57C>T |  | 1 |
| W198*, c.-81C>T, | c.-52C>T |  | 1 |
| W351*, | c.-54C>T |  | 1 |
| L164R; | c.-48G>A |  | 1 |
| L298W; |  | c.-15C>T | 1 |
| L384P; | c.G-51A; |  | 1 |
| K152Q;, | c.-48G>A | c.-15C>T | 1 |
| M377I;T579A; |  |  | 1 |
| F183L; | 88insAT |  | 1 |
| F408S;G268V; |  |  | 1 |
| P232A; |  |  | 1 |
| P232R; |  |  | 1 |
| P232S; |  |  | 1 |
| P232S; | c.-81C>T |  | 1 |
| P286H;T394A; | c.-48G>A, |  | 1 |
| P501Q; |  |  | 1 |
| S315R; |  |  | 1 |
| S315N; | c.G-51A; |  | 1 |
| S315N;I317V; |  |  | 1 |
| S315N;I82V; |  |  | 1 |
| S315G;; | c.G-51A; | c.-8T>C | 1 |
| S315I; |  |  | 1 |
| S315T; | c.-48G>A, |  | 1 |
| S315T; | G-189C; |  | 1 |
| S315T; | G-451A; |  | 1 |
| S315T; | G-515A; |  | 1 |
| S315T; | G-645T; |  | 1 |
| S315T; | T-311C; |  | 1 |
| S315T; | T-436C; |  | 1 |
| S315T; | A-369C; |  | 1 |
| S315T;A15P; |  |  | 1 |
| S315T;D419E; |  |  | 1 |
| S315T; | C-52A; |  | 1 |
| S315T; |  | c.-15C>T, | 1 |
| S315T; |  | c.-15C>T, | 1 |
| S315T;, | G-158C; | c.-15C>T | 1 |
| S315T; |  | c.-17G>T | 1 |
| S315T; |  | c.-8T>A | 1 |
| S315T;E195D; |  |  | 1 |
| S315T;; |  | I122V | 1 |
| S315T; |  | S94A | 1 |
| T271P; | c.-74G>A |  | 1 |
| W149G; | c.-15C>T |  | 1 |
| W505C; |  |  | 1 |
| W728C; |  |  | 1 |
| Y155C; |  | c.-15C>T | 1 |
| Y155C;, |  | c.-15C>T | 1 |
| Y155C;E3Q; |  | c.-15C>T | 1 |
| Y337C;; | c.G-51A |  | 1 |
| Y413C; | C-52A; | c.-15C>T | 1 |
| Y98C; |  |  | 1 |
| Y98C; | c.-52C>T |  | 1 |
| V196A; |  | c.-15C>T;I21V | 1 |
| V1A; |  | c.-15C>T | 1 |
| V697L; |  |  | 1 |
| V697L;; |  | c.-15C>T;A239T; | 1 |
| V697L;T625P; |  | c.-15C>T | 1 |
| WT |  |  | 29 |
| Total |  |  | 546 |

| **Table S6 The distribution of gene mutations associated with ethambutol resistance.** | | | |
| --- | --- | --- | --- |
| *embB* mutation  (codon position) | *embA* mutation  (codon position) | *embC* mutation  (codon position) | No. of isolates with  resistant phenotype |
| M306V; |  |  | 63 |
| Q497R |  |  | 32 |
| M306I |  |  | 29 |
| M306V;I563L; |  |  | 11 |
| G406A; |  |  | 7 |
| G406D; |  |  | 5 |
| M306L; |  |  | 5 |
| G406S; |  |  | 4 |
| M306I;G406D; |  |  | 4 |
| M306V; | c.-16C>T |  | 4 |
| D328Y; |  |  | 3 |
| Q497K; |  |  | 3 |
| Y319S; |  |  | 3 |
| G406S;T141R; |  |  | 2 |
| M306I;G406S; |  |  | 2 |
| M306V;D1024N |  |  | 2 |
| M306V; | c.-16C>G, |  | 2 |
| M306V; |  | V974L; | 2 |
| M306V; | E951D |  | 2 |
| M306V;G406D |  |  | 2 |
| D1024N; | T652R |  | 1 |
| D1024N;G406V; |  |  | 1 |
| D354A;D1024N |  |  | 1 |
| D354A |  |  | 1 |
| D354A;M306V |  |  | 1 |
| D354A;T141R; | c.-12C>T |  | 1 |
|  | c.-16C>T |  | 1 |
|  |  | T100S; | 1 |
| Q497R;M306I; | A291T |  | 1 |
| Q497R;Y334H; |  |  | 1 |
| Q497P;D354A | A835T |  | 1 |
| Q853E; |  |  | 1 |
| E405D; |  |  | 1 |
| G406A;D328H; |  |  | 1 |
| G406A; | c.-12C>T |  | 1 |
| G406A;I563L; |  |  | 1 |
| G406D;D328G |  |  | 1 |
| G406D;Q853H; |  |  | 1 |
| G406C; |  |  | 1 |
| G406S; |  | V974L; | 1 |
| G406S; | E951D |  | 1 |
| G406V; |  |  | 1 |
| I563L;G406S; |  |  | 1 |
| M1000R; |  |  | 1 |
| M306I;D1024N |  |  | 1 |
| M306I; | c.-11C>A | V974L; | 1 |
| M306I; | c.-16C>G |  | 1 |
| M306I; | c.-16C>T |  | 1 |
| M306I; | E951D |  | 1 |
| M306I; | G5V |  | 1 |
| M306I; | I914V | V627I; | 1 |
| M306I; | L653P |  | 1 |
| M306I; | P2T |  | 1 |
| M306I;Q497R |  |  | 1 |
| M306I;Q497K; |  |  | 1 |
| M306I;S66R | c.-12C>T |  | 1 |
| M306I;V130A; |  |  | 1 |
| M306L; | c.-12C>T |  | 1 |
| M306L;G246R; |  |  | 1 |
| M306L;I563L; |  |  | 1 |
| M306T; |  |  | 1 |
| M306V; | R11Trp |  | 1 |
| M306V; | R380L |  | 1 |
| M306V; | c.-16C>G |  | 1 |
| M306V; |  | A418T; | 1 |
| M306V; |  | G780D; | 1 |
| M306V;Q855H; |  |  | 1 |
| M306V;S347T; |  |  | 1 |
| M306V;T643I; |  |  | 1 |
| M306V;T643I;L391F; |  |  | 1 |
| T1084P; |  |  | 1 |
| Y319D |  |  | 1 |
| Y319C;.M306V | c.-12C>T |  | 1 |
| Y319C; | c.-12C>T |  | 1 |
| Y319C; |  |  | 1 |
| Y319C; | E951D; |  | 1 |
| V130A; |  |  | 1 |
| WT |  |  | 12 |
| Total |  |  | 256 |

| **Table S7. The distribution of gene mutations associated with moxifloxacin resistance.** | | | |
| --- | --- | --- | --- |
| *gyrA* mutation  (codon position) | | *gyrB* mutation  (codon position) | No. of isolates with  resistant phenotype |
| E21Q;G668D; | D94G; |  | 62 |
| E21Q;G668D;;S95T; | A90V; |  | 43 |
| E21Q;G668D;S95T; |  |  | 22 |
| E21Q;G668D; | D94A; |  | 14 |
| E21Q;G668D; | D94Y; |  | 10 |
| E21Q;G668D; | D94N; |  | 8 |
| E21Q;G668D;S95T; | D94G; |  | 6 |
| E21Q;G668D;S95T; | S91P; |  | 5 |
| E21Q;G668D;S95T; | D94Y; |  | 4 |
| E21Q;G668D;S95T; | A90V; | D461N; | 3 |
| E21Q;G668D; | D94H; |  | 3 |
| E21Q;G668D;S95T; |  | D461N; | 3 |
| E21Q;G668D; | D94G;A74S; | M100V; | 2 |
| E21Q;G668D; | D94G; | F396L; | 2 |
| E21Q;G668D;S95T; | A90V;R448C; |  | 2 |
| E21Q;G668D;S95T; | A90V;G236V; |  | 2 |
| E21Q;G668D;S95T; | A90V; | G512R; | 2 |
| E21Q;G668D; | D94A;A90V; |  | 2 |
| E21Q;G668D; | D94N; | D645A; | 2 |
| E21Q;G668D;S95T; | D89N; |  | 2 |
| E21Q;G668D; | A90V;D94G; |  | 1 |
| E21Q;G668D; | D94G; | A504T; | 1 |
| E21Q;G668D; | D94G; | A504V; | 1 |
| E21Q;G668D; | D94G; | A597T; | 1 |
| E21Q;G668D; | D94G; | G512R; | 1 |
| E21Q;G668D; | D94G;L653V; |  | 1 |
| E21Q;G668D;S95T; | A90V;D94G; |  | 1 |
| E21Q;G668D;S95T; | A90V; | T500N; | 1 |
| E21Q;G668D; | D94Y;D94G; |  | 1 |
| E21Q;G668D; | D94Y; | G512R; | 1 |
| E21Q;G668D; | D94Y; | T167M; | 1 |
| E21Q;G668D; | D94A;A74S; |  | 1 |
| E21Q;G668D; | D94A;D94G; |  | 1 |
| E21Q;G668D; | D94A; | A504T; | 1 |
| E21Q;G668D; | D94A; | G512R; | 1 |
| E21Q;G668D; | D94A; | T500N; | 1 |
| E21Q;G668D; | D94A; | V427M; | 1 |
| E21Q;G668D; | D94A;S91P; |  | 1 |
| E21Q;G668D; | D94N;D94G; |  | 1 |
| E21Q;G668D;S95T; | S91P;D94A; |  | 1 |
| E21Q;G668D;S95T; | S91P; |  | 1 |
| E21Q;G668D;S95A; |  |  | 1 |
| E21Q;G668D;S95T; | D94A; |  | 1 |
| E21Q;G668D;S95T; | D94G; | A597T; | 1 |
| E21Q;G668D;S95T; | G88C; |  | 1 |
| E21Q;G668D;S95T; |  | A504V; | 1 |
| E21Q;G668D;S95T; |  | R446C; | 1 |
| E21Q;G668D;S95T; | L455V; | R446L; | 1 |
| E21Q;G668D;S95T; | S91P;A288D; |  | 1 |
| E21Q;S95T; |  |  | 1 |
| Total |  |  | 229 |

| **Table S8. The distribution of gene mutations associated with levofloxacin resistance.** | | | |
| --- | --- | --- | --- |
| *gyrA* mutation  (codon position) | | *gyrB* mutation  (codon position) | No. of isolates with  resistant phenotype |
| E21Q;G668D; | D94G; |  | 62 |
| E21Q;G668D; | A90V;S95T; |  | 43 |
| E21Q;G668D; | S95T; |  | 16 |
| E21Q;G668D; | DS94AT; |  | 14 |
| E21Q;G668D; | D94Y; |  | 10 |
| E21Q;G668D; | DS94NT; |  | 8 |
| E21Q;G668D; | S95T;D94G; |  | 6 |
| E21Q;G668D; | S95T;S91P; |  | 5 |
| E21Q;G668D; | S95T;D94Y; |  | 4 |
| E21Q;G668D; | A90V;S95T; | D461N; | 3 |
| E21Q;G668D; | DS94HT; |  | 3 |
| E21Q;G668D; | S95T; | D461N; | 3 |
| E21Q;G668D;A74S; | D94G; | M100V; | 2 |
| E21Q;G668D; | D94G; | F396L; | 2 |
| E21Q;G668D;R448C; | A90V;S95T; |  | 2 |
| E21Q;G668D;G236V; | A90V;S95T; |  | 2 |
| E21Q;G668D; | A90V;S95T; | G512R; | 2 |
| E21Q;G668D; | DS94AT;A90V; |  | 2 |
| E21Q;G668D; | DS94NT; | D645A; | 2 |
| E21Q;G668D;D89N; | S95T; |  | 2 |
| E21Q;G668D; | A90V;D94G; |  | 1 |
| E21Q;G668D; | D94G; | A504T; | 1 |
| E21Q;G668D; | D94G; | A504V; | 1 |
| E21Q;G668D; | D94G; | A597T; | 1 |
| E21Q;G668D; | D94G; | G512R; | 1 |
| E21Q;G668D;L653V; | D94G; |  | 1 |
| E21Q;G668D; | A90V;S95T;D94G; |  | 1 |
| E21Q;G668D; | A90V;S95T; | T500N; | 1 |
| E21Q;G668D; | D94Y;D94G; |  | 1 |
| E21Q;G668D; | D94Y; | G512R; | 1 |
| E21Q;G668D; | D94Y; | T167M; | 1 |
| E21Q;G668D;A74S; | DS94AT |  | 1 |
| E21Q;G668D; | DS94AT;D94G; |  | 1 |
| E21Q;G668D; | DS94AT; | A504T; | 1 |
| E21Q;G668D; | DS94AT; | G512R; | 1 |
| E21Q;G668D; | DS94AT; | T500N; | 1 |
| E21Q;G668D; | DS94AT; | V427M; | 1 |
| E21Q;G668D; | DS94AT;S91P; |  | 1 |
| E21Q;G668D; | DS94NT;D94G; |  | 1 |
| E21Q;G668D; | S91P;S95T;DS94AT; |  | 1 |
| E21Q;G668D; | S91P;S95T; |  | 1 |
| E21Q;G668D; | S95T;D94G; | A597T; | 1 |
| E21Q;G668D; | S95T;DS94AT; |  | 1 |
| E21Q;G668D;G88C; | S95T; |  | 1 |
| E21Q;G668D;A288D; | S95T;S91P; |  | 1 |
| E21Q; | S95T; |  | 1 |
| E21Q;G668D; | S95T; | L455V; | 1 |
| E21Q;G668D; | S95T; | S91P;A288D; | 1 |
| Total |  |  | 221 |

| **Table S9 The distribution of gene mutations associated with amikacin resistance.** | | | |
| --- | --- | --- | --- |
| *rrs* muation | *eis* mutaion | *whiB7* mutaion | No. of isolates with  resistant phenotype |
| A1401G; |  |  | 38 |
| G1484T; |  |  | 4 |
| A1401G; | V163I; |  | 8 |
|  | V163I; |  | 1 |
| WT |  |  | 9 |
| Total |  |  | 60 |

| **Table S10 The distribution of gene mutations associated with kanamycin resistance.** | | | |
| --- | --- | --- | --- |
| *rrs* muation | *eis* mutaion | *whiB7* muation | No. of isolates with  resistant phenotype |
| A1401G; |  |  | 48 |
| G1484T; |  |  | 4 |
|  | c.-10G>A |  | 5 |
| WT |  |  | 11 |
| Total |  |  | 68 |

| **Table S11 The distribution of gene mutations associated with ethionamide resistance.** | | | |
| --- | --- | --- | --- |
| *inhA* mutaion | *ethA* muation | *fabG1* mutaion | No. of isolates with resistant phenotype |
|  |  | C-15T; | 19 |
|  | L35R |  | 15 |
|  | c.110_110del | T-8C; | 2 |
|  | c.110_110del; | C-15T; | 3 |
|  | F48S | C-15T; | 2 |
|  | S208* | C-15T | 2 |
|  | S266R;c.1431_1431del; | T-8C; | 2 |
|  | S266R;P454L | C-15T; | 2 |
|  | R292* |  | 1 |
|  | c.1047_1047del, | C-15T; | 1 |
|  | c.1058_1061del |  | 1 |
|  | c.110_110del |  | 1 |
|  | c.1371_1372insCCTG; |  | 1 |
|  | c.1391_1392insGA |  | 1 |
|  | c.672_673insG |  | 1 |
|  | c.706_707insA |  | 1 |
|  | c.752_753insG |  | 1 |
|  | c.752_753insG; | T-8A; | 1 |
|  | c.752_753insG; | T-8C; | 1 |
|  | c.819_819del, | C-15T; | 1 |
|  | c.83_83del; |  | 1 |
|  | c.839_839del | T-8C | 1 |
|  | c.9_9del |  | 1 |
|  | c.938_939insT; |  | 1 |
|  | Chromosome:g.4326004_4326447del |  | 1 |
|  | Chromosome:g.4327059_4327128del; | C-15T; | 1 |
|  | Q254P; |  | 1 |
|  | Q254P | C-15T; | 1 |
|  | Q271* |  | 1 |
|  | Q347* |  | 1 |
|  | E3fs |  | 1 |
|  | G275E |  | 1 |
|  | I161V | C-15T | 1 |
|  | L190P | C-15T | 1 |
|  | L194R | C-15T | 1 |
|  | L194Q |  | 1 |
|  | L23R | G-17T | 1 |
|  | L35R | G-32T | 1 |
|  | L35R | T-8C | 1 |
|  | L393P |  | 1 |
|  | F100fs | C-15T | 1 |
|  | P164L |  | 1 |
|  | P334A |  | 1 |
|  | P334A | C-15T | 1 |
|  | P68L | T-8C | 1 |
|  | S266R |  | 1 |
|  | S266R;c.1054_1054del; |  | 1 |
|  | S266R;c.1205_1206insGT; | C-15T; | 1 |
|  | S266R;c.364_364del; | T-8C; | 1 |
|  | S266R;c.672_673insG |  | 1 |
|  | S266R,c.1431_1431del | T-8C; | 1 |
|  | S266R;L194_A195delinsP;; |  | 1 |
|  | S266R;P164Q |  | 1 |
|  | S266R;S55P | T-8C | 1 |
|  | T203P; | C-15T | 1 |
|  | T383P |  | 1 |
|  | W116R |  | 1 |
|  | V296Met | C-15T; | 1 |
| A239T | S266R;P454L | C-15T; | 1 |
| I194T |  | C-15T; | 1 |
| I21T |  | C-15T | 1 |
| I21T | Lys280R | C-15T; | 1 |
| I21V |  | C-15T | 1 |
| I95L |  | C-15T | 1 |
| S94A |  | C-15T | 1 |
| S94A | G11S |  | 1 |
| V92Met |  | C-15T; | 1 |
|  | WT |  | 10 |
| Totol |  |  | 116 |
