# Supplementary material for: Insight into the drug-resistant characteristics and genetic diversity of multidrug-resistant Mycobacterium tuberculosis in China
Source: Microbiol Spectr. 2023 Sep 21;11(5):e01324-23. doi: 10.1128/spectrum.01324-23 (PMC10581218; doi:10.1128/spectrum.01324-23)
Supplement: Figure S1 — The MIC distribution of the 546 MDR isolates against bedaquiline, clofazimine, delamanid, and linezolid. Purple indicates drug-resistant strains with MIC greater than the breakpoint. [file spectrum.01324-23-s0002.pdf]

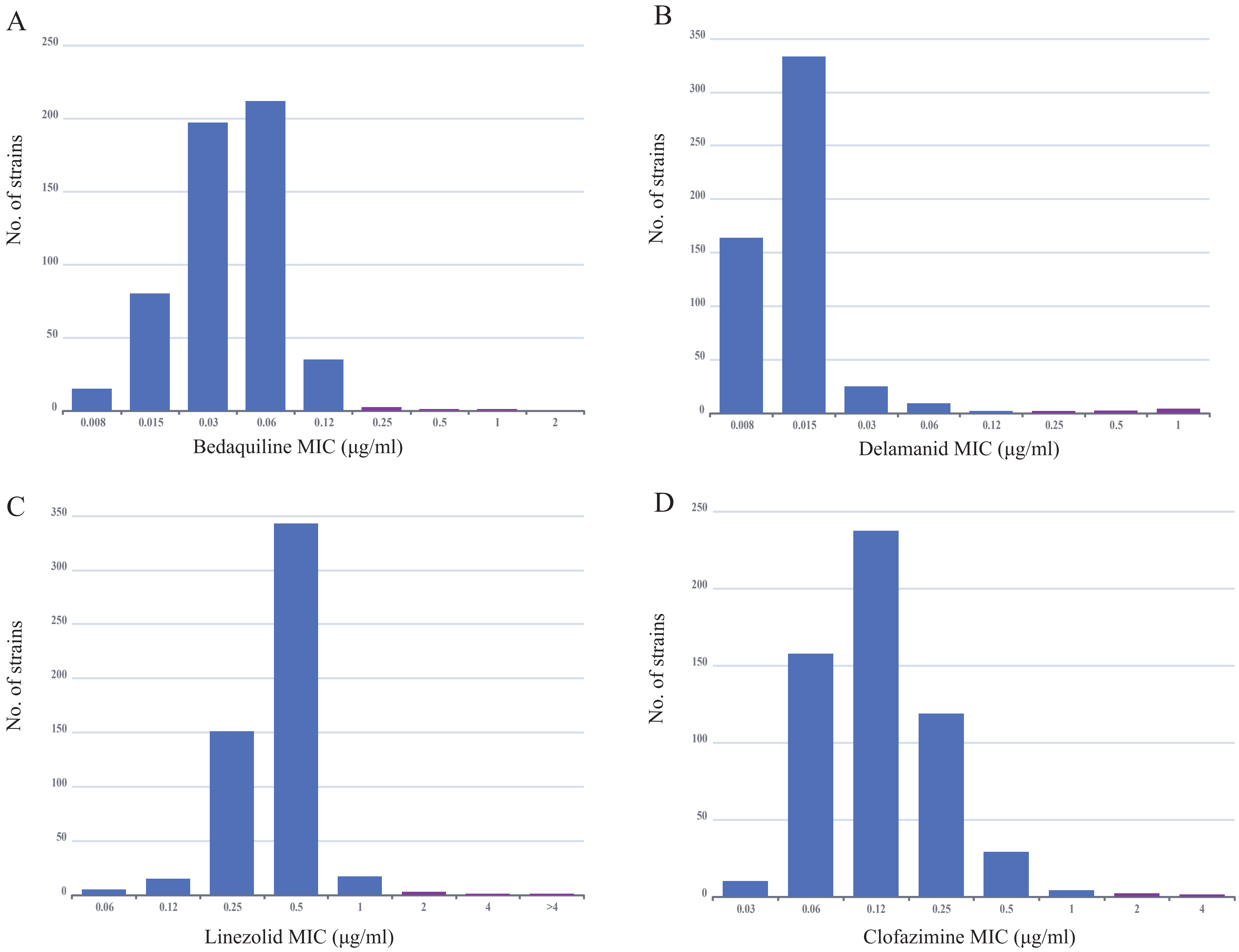

Figure S1. The MIC distribution of the 546 MDR isolates against bedaquiline, clofazimine, delamanid and linezolid. Purple indicates drug-resistant strains with MIC greater than the breakpoint.
